# Prevalence and Mortality of Individuals With X-Linked Hypophosphatemia: A United Kingdom Real-World Data Analysis

**DOI:** 10.1210/clinem/dgz203

**Published:** 2019-11-15

**Authors:** Samuel Hawley, Nick J Shaw, Antonella Delmestri, Daniel Prieto-Alhambra, Cyrus Cooper, Rafael Pinedo-Villanueva, M Kassim Javaid

**Affiliations:** 1 Nuffield Department of Orthopaedics, Rheumatology and Musculoskeletal Sciences, University of Oxford, Oxford, UK; 2 Birmingham Women’s and Children’s Hospital NHS Foundation Trust, Birmingham, UK; 3 Institute of Metabolism & Systems Research, University of Birmingham, Birmingham, UK; 4 GREMPAL Research Group, Idiap Jordi Gol and CIBERFes, Universitat Autònoma de Barcelona and Instituto de Salud Carlos III, Barcelona, Spain; 5 MRC Lifecourse Epidemiology Unit, University of Southampton, Southampton, UK

**Keywords:** XLH, hypophosphatemia, rickets, prevalence, survival, rate

## Abstract

**Background:**

X-linked hypophosphatemia (XLH) is a rare multisystemic disease with a prominent musculoskeletal phenotype. We aim here to improve understanding of the prevalence of XLH across the life course and of overall survival among people with XLH.

**Methods:**

This was a population-based cohort study using a large primary care database in the United Kingdom (UK) from 1995 to 2016. XLH cases were matched by age, gender, and practice to up to 4 controls. Trends in prevalence over the study period were estimated (stratified by age) and survival among cases was compared with that of controls.

**Findings:**

From 522 potential cases, 122 (23.4%) were scored as at least possible XLH, while 62 (11.9%) were classified as highly likely or likely (conservative definition). In main analyses, prevalence (95% CI) increased from 3.1 (1.5–6.7) per million in 1995–1999 to 14.0 (10.8–18.1) per million in 2012–2016. Corresponding estimates using the conservative definition were 3.0 (1.4–6.5) to 8.1 (5.8–11.4). Nine (7.4%) of the possible cases died during follow-up, at median age of 64 years. Fourteen (2.9%) of the controls died at median age of 72.5 years. Mortality was significantly increased in those with possible XLH compared with controls (hazard ratio [HR] 2.93; 95% CI, 1.24–6.91). Likewise, among those with likely or highly likely XLH (HR 6.65; 1.44–30.72).

**Conclusions:**

We provide conservative estimates of the prevalence of XLH in children and adults within the UK. There was an unexpected increase in mortality in later life, which may have implications for other fibroblast growth factor 23–related disorders.

X-linked hypophosphatemic rickets (XLH) is a rare multisystemic disease of mineral homeostasis that has a prominent skeletal phenotype characterized by renal phosphate wasting due to mutations in the *PHEX* gene (1). It is the most common form of heritable rickets (2). The key molecular mechanism involves excess fibroblast growth factor 23 (FGF23) production, a phosphatonin first identified in autosomal dominant hypophosphatemic rickets (3) and tumor-induced osteomalacia (4, 5). XLH usually manifests early in life with shortened height and bowing of the legs, and while these can be improved with pharmacotherapy, they likely persist into adulthood along with increased risk of fractures, arthritis, dental abscesses, and enthesopathy (calcification of tendons and ligaments) (2, 6).

Traditional therapy for XLH includes activated vitamin D and oral phosphate, which while effective in increasing childhood growth, the therapy is poorly tolerated and of unknown efficacy in adults with XLH (7). Burosumab, a neutralizing antibody of FGF23, increases serum phosphorus and improves rickets and linear growth (8). In adults, burosumab also significantly improves serum phosphate as well as fracture healing, pain, and stiffness (9). However, this is a relatively novel therapy, and, in many countries, policy makers are unclear about which adults should be eligible for therapy. This is compounded by the scarcity of data on the prevalence and outcomes of adults with XLH.

Three previous studies of the prevalence of XLH in children have used a mixture of hospital surveys and registry data with conflicting prevalence rates (10–12), due in part to differences in criteria for case identification and validation. Accurate data for adults is compounded by the lack of any standard management for adults with XLH in terms of monitoring laboratory values, skeletal status, and other characteristics. Hence, it is possible that in the United Kingdom (UK), most adults are managed principally in the primary care setting. The National Health Service (NHS) healthcare system within the UK has near universal coverage and represents an opportune data resource to explore the prevalence of a rare disease such as XLH and its associated mortality rate. Our aim was to determine the prevalence of XLH in both children and adults and to describe survival across the life course using routinely collected medical data.

## Methods

### Study design and participants

This UK-based study used primary care health data obtained from the UK Clinical Practice Research Datalink (CPRD) GOLD dataset from 1995 to 2016. As of 2013, CPRD GOLD covered more than 11.3 million patients from 674 general practitioner (GP) practices and had a representative coverage of approximately 7% of the UK population (13). Only GP practices that successfully complete the up to standard process are then included in the CPRD GOLD dataset. CPRD uses the Read code system, devised by Dr Read, a UK GP who pioneered in data coding(14), and includes more than 100 000 codes for clinical events in primary care (15). Mortality data for England and Wales were also obtained from linkage to the Office for National Statistics (ONS) dataset which is considered the gold standard for mortality data. Date of death was therefore based on ONS data for the 60% of GP practices where this linkage was available and of sufficient quality, otherwise death date as recorded by CPRD was used (which has been shown to be comparable with the ONS(14)).

There is no agreed algorithm for identifying cases with XLH using real world data. Methods from previous studies(10–12) were modified by the availability of data in the CPRD by 2 experts in familial hypophosphatemia in children (NS) and adults (MKJ). A list of potential diseases that cause rickets/osteomalacia and could represent XLH was used to extract potential cases and their controls from the CPRD dataset (Table 1). Next, an algorithm was developed to grade cases (“highly likely,” “likely,” “possible,” “unlikely” or “unable to determine”) using other primary care data that included:

**Table 1.** List of Read Codes Used to Identify Individuals With a Potential Diagnosis of XLH: Stratified by Final Grading for Likelihood of XLH

Read Code	Read Code Label	Frequency						PPV (%)
		Highly likely	Likely	Possible/ potential	Unlikely	Unable to determine	TOTAL	
2374	O/E —rickety rosary	0	0	0	4	2	6	0
C280.00	Active rickets	0	0	0	9	8	17	0
C281.00	Late effect of rickets	0	1	0	4	0	5	20
C282.00	Osteomalacia, unspecified	0	0	3	75	15	93	0
C28A.00	Vitamin D dependent rickets	0	0	0	8	0	8	0
C353000	Hypophosphatasia	3	6	45	129	57	240	3.8
C353100	Hypophosphatasia rickets	3	10	1	6	8	28	46.4
C353200	Vitamin-D-resistant rickets	3	3	2	9	9	26	23.1
C353211	Hypophosphatemic rickets	17	17	5	15	13	67	50.7
C353700	X-linked Hyposphataemic rickets	1	0	1	0	0	2	50.0
C353800	Autosomal dominant hypophosphatemic rickets	0	0	0	1	1	2	0
C353z00	Disorders of phosphorus metabolism NOS	0	0	0	11	6	17	0
K080300	Renal rickets	0	0	1	6	4	11	0
ALL		27	37	58	277	123	522	

Abbreviations: O/E, on examination; PPV, positive predictive value (using highly likely and likely as reference standard).

(a) Terms for other diagnoses that could mimic XLH, such as cystinosis or fibrous dysplasia (Supplementary Item 1) (16),
(b) Laboratory tests performed in the primary care setting including counts of: normal, low, and very low (< 75% of lower limit of normal range) serum phosphate; normal or high, low alkaline phosphatase (ALP); normal, low, and high serum adjusted calcium; normal or high serum parathyroid (PTH); estimated glomerular filtration rate (eGFR) < 15 mL/min/m^2^ or < 30–15mL/min/m^2^; normal or high alanine transferase (ALT) values; normal or low serum bicarbonate values. Existing CPRD reference ranges for laboratory values were used to estimate values for the 20% of patients missing these ranges (Supplementary Item 2) (16).
(c) Prescriptions for more than 12 months of any activated vitamin D preparation, oral phosphate preparations, bicarbonate, potassium or calcium supplements (see Supplementary Item 3 for drug codes) (16).

A key feature of XLH is low serum phosphate. From the extracted cases, individuals with 2 or more low phosphate values or 50% or more low phosphate values if fewer than 3 values were recorded or no phosphate results recorded were selected for grading. Major criteria, unexpected criteria, and red flags were developed. Major criteria were high ALP, or more than 1 year of activated vitamin D therapy or phosphate therapy. Unexpected criteria included less than 50% serum phosphate values below the minimum reference range, presence of low ALP values or only normal ALP values, or a low adjusted serum calcium or bicarbonate value. Criteria that would discount a diagnosis of XLH were only low ALP values, 2 or more low adjusted calcium values, 2 or more low bicarbonate values, treatment with bicarbonate or potassium supplements or a competing diagnosis. NS and MKJ independently graded cases using these criteria (Supplementary Item 4) (16). Patients with no recorded phosphate values and no unexpected or red flags were designated as not able to be graded.

Given the expected excess of females with XLH, graders were blinded to the gender of potential patients. After the first round of grading, a more detailed record was created for each patient discordantly graded highly likely/likely versus other grades in a second round with the complete clinical record made available. In a post hoc analysis we explored the presence of Read codes for renal stones and/or hypercalciuria (codes in Supplementary Item 3 (16)).

A physician verification questionnaire (Supplementary Item 5 (16) was also sent to GPs of eligible patients with potential diseases that cause rickets/osteomalacia (codes included in Table 1) and who were still alive and in a practice that agreed to participate in a survey consisting of 4 questions. The questionnaire was sent twice with a reminder by CPRD.

Up to 4 non-XLH controls (without any of the Read codes of interest) of same age, gender, and GP practice were matched to each potential XLH case. Data on demographics, prescriptions, laboratory test results, and comorbidities were extracted from CPRD GOLD for both potential cases and controls.

### Statistical analysis

To evaluate the reproducibility of the XLH likelihood grading by the 2 clinicians, a weighted Kappa was calculated to assess concordance across the 5 different categories (after both the first and second round of grading). Descriptive statistics (counts and percentages) were used to assess agreement between final likelihood gradings and returned GP questionnaires. Temporal trends in XLH prevalence and associated 95% confidence intervals (CI) were estimated by dividing mid-year counts of XLH cases by CPRD annual denominator data. Prevalence estimates were generated using all included cases and stratified by age in order to explore prevalence in childhood vs adulthood: younger than 16 years vs 16 years and older. Annual estimates were depicted graphically, and estimates reported separately for the first 5 years of the study period (1995–1999) versus last 5 years (2012–2016).

An index date for each XLH patient was defined as the first relevant Read code date after the GP practice of registration was flagged as “up to standard” by CPRD for clinical research. Index dates for controls were anchored to match those of cases. Patients were followed from index date until the earliest of: death, other types of transfer out of practice, or end of study. Deaths (and causes where available) were then identified and mortality incidence rates calculated separately for cases and controls. Kaplan-Meier estimates were plotted following univariable extended Cox regression using patient age as the time axis (17).

Prevalence and mortality analyses were repeated using only cases (and their matched controls) graded as very likely or likely, to assess if the main findings persisted when using a conservative case definition, albeit with a smaller sample size. A further sensitivity analysis was performed for the mortality analysis, in which eligible patients in England and Wales were followed from index date until the earliest of death date or data extraction date (irrespective of transference out of practice and last collection date in CPRD) in order to make use of the continued linkage to ONS data.

### Role of the funding source and ethics committee approval

The funder of the study had no role in the study design, data collection, data analysis, data interpretation, or writing of the report. The corresponding author had full access to all of the data in the study and had final responsibility for the decision to submit for publication. The Independent scientific Advisory Committee (ISAC) approved the study and since only de-identifiable routinely-collected data was used, no further approvals were required.

## Results

A total of 522 patients were identified using the initial CPRD codes. Following the first round of independent grading of potential cases, the weighted Kappa for inter-grader agreement was 0.88 (95% CI, 0.85–0.91). Following independent re-grading of discordant cases, it increased to 0.92 (95% CI, 0.89–0.94) when using 5 likelihood categories and to 0.98 (95% CI, 0.96–1.00) when using 2 categories (likely and very likely vs other grades).

Of the 522 initially identified potential cases, 122 were used in main analyses: 27 highly likely, 37 likely and 58 possible (Table 2). An expected preponderance of females (70%), childhood diagnoses (median age at earliest Read code < 10 years) and phosphate supplementation (> 95%) was observed amongst the very likely and likely cases, although not in the “possible” ones (Table 2 and Supplementary Item 6) (16).

**Table 2.** Patient Characteristics of Initial Sample of 522 Potential XLH cases: Stratified by Final Grading for Likelihood of XLH (1995–2016)

	Grading					TOTAL
	Highly Likely	Likely	Possible	Unlikely	Unable to Determine	
n	27	37	58	277	123	522
Female, n (%)	19 (70.4)	26 (70.3)	24 (41.4)	159 (57.4)	46 (37.4)	274 (52.5)
Median age at earliest Read code, years (IQR)	7 (1–25)	3 (1–7)	47.5 (26–61)	37 (18–57)	2 (0–27)	29 (3–52)
Median duration of follow-up, years (IQR)	8.28 (5.40–14.01)	6.36 (2.29–10.01)	2.88 (1.15–5.53)	3.99 (1.43–9.64)	1.64 (0.47–4.92)	3.51 (1.29–8.30)
Prior hypercalciuria or renal stones, n (%)	2 (7.4)	0 (0)	0 (0)	15 (5.4)	1 (0.8)	18 (3.5)
Low serum phosphate count, n (%)	19 (70.4)	11 (29.7)	49 (84.5)	63 (22.7)	9 (7.3)	151 (28.9)
High alkaline phosphatase count, n (%)	4 (14.8)	3 (8.1)	3 (5.2)	24 (8.7)	1 (0.8)	35 (6.7)
Activated vitamin D therapy (ever initiated), n (%)	19 (70.4)	6 (16.2)	12 (20.7)	68 (24.6)	2 (1.6)	107 (20.5)
Activated vitamin D therapy duration, years (IQR)^*a*^	4 (14.8)	3 (8.1)	12 (20.7)	49 (17.7)	3 (2.4)	71 (13.6)
Phosphate supplements (ever initiated), n (%)	27 (100)	35 (95.6)	9 (15.5)	49 (17.7)	10 (8.1)	130 (24.9)
Phosphate supplements duration, years (IQR)^*b*^	8.56 (5.02–17.21)	8.30 (3.17–11.07)	1.29 (0.55–7.42)	4.04 (1.98–7.45)	-	5.35 (2.38–11.08)

Abbreviations: IQR, interquartile range.

^*a*^23 Patients on activated vitamin D unable to determine duration given missing dates.

^*b*^16 patients on phosphate supplements unable to determine duration given missing dates.

From the GP verification, there were 69/106 (65%) questionnaires returned, of which a total of 17 (25%) reported a definite/possible familial hypophosphatemic condition (results shown in Supplementary Item 7) (16).

### Prevalence

**Figure 1.**
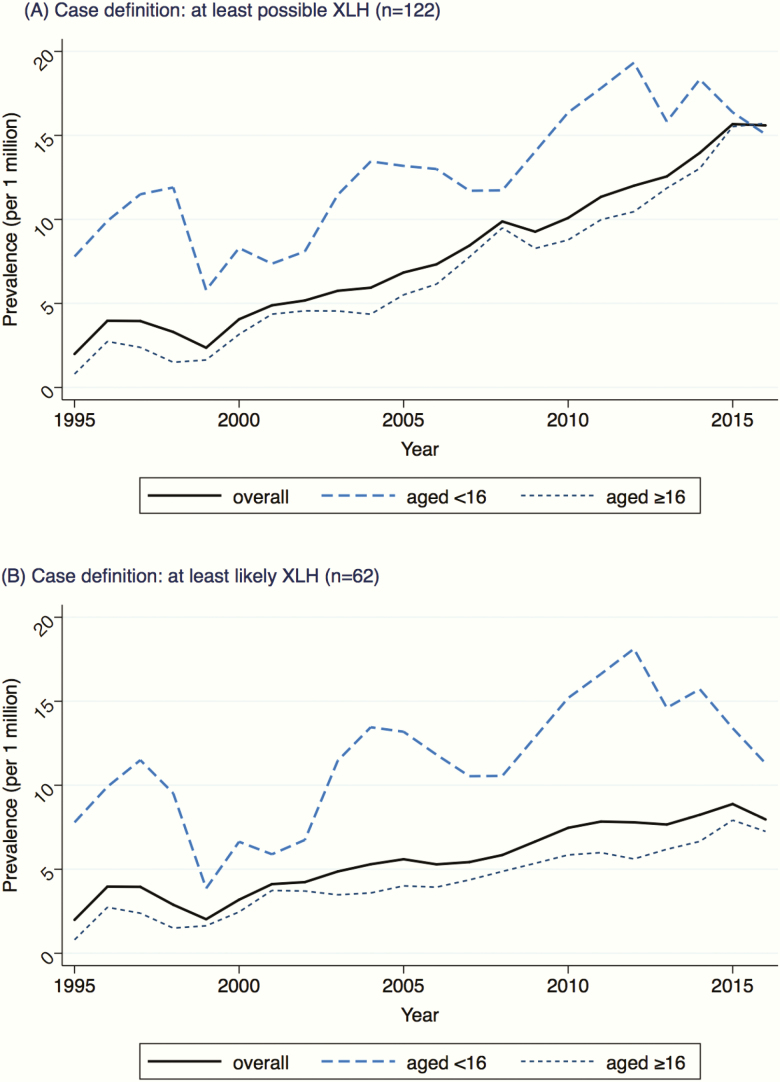
Prevalence of XLH by calendar year and age band for: (A) all possible cases of XLH and (B) only likely and highly likely cases of XLH.

In the main analysis, prevalence of XLH increased from 3.1 (95% CI, 1.5–6.7) per million in the first 5 years of the study (1995–1999) to 14.0 (95% CI, 10.8–18.1) per million in the last 5 years (2012–2016), (Fig. 1A). Likewise, prevalence per million increased in both pediatric (9.4 [95% CI, 3.2–27.2] to 17.0 [95% CI, 9.7–29.6]) and adult (1.8 [95% CI, 0.6–5.4] to 13.3 [95% CI, 9.9–17.9]) populations over the study period. When using the more conservative sensitivity definition of only highly likely and likely cases, prevalence estimates increased from 3.0 (95% CI, 1.4–6.5) per million in the first 5 years to 8.1 (95% CI, 5.8–11.4) per million in the last 5 years of the study period, Fig. 1B. As per main analyses, prevalence per million increased in both pediatric (8.5 [95% CI, 2.7–26.4] to 14.6 [95% CI, 8.1–26.6]) and adult (1.8 [95% CI, 0.6–5.4] to 6.7 [95% CI, 4.5–10.2]) populations.

### Mortality data

**Figure 2.**
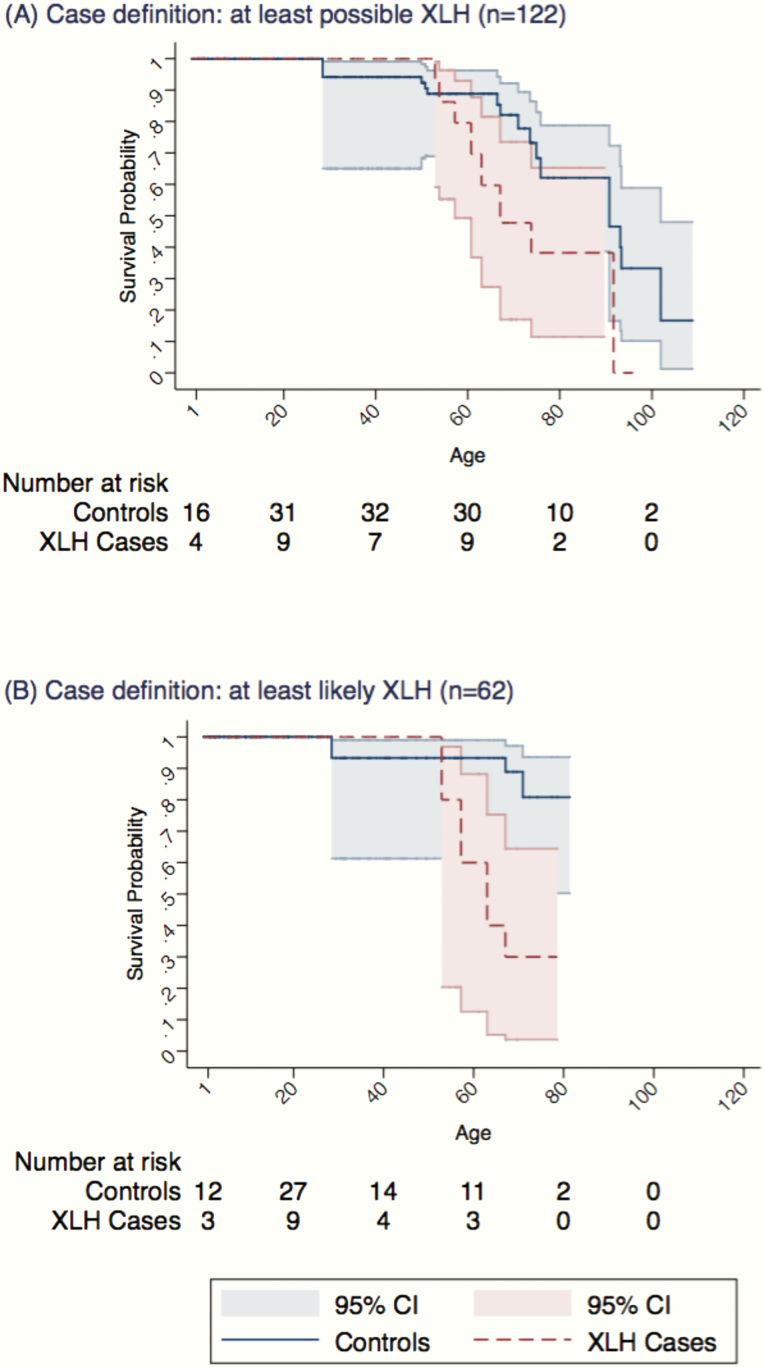
Kaplan-Meier plot for survival across lifespan for: (A) all possible cases of XLH and (B) only likely and highly likely cases of XLH.

Of the 610 patients included in the Cox model, 9 (7.4%) of the 122 cases died during a median of 4.6 (interquartile range [IQR], 2.1–8.6) years of follow-up, at a median age of 64 (IQR, 58–74) years. This equated to a rate of 12.1 (95% CI, 6.3–23.3)/1000 person-years. Fourteen (2.9%) controls died during a median of 4.2 (IQR, 2.84–8.09) years of follow-up, at median age of 72.5 (IQR, 29–71) years. This equated to a rate of 4.8 (95% CI, 2.8–8.1)/1000 person-years and a hazard ratio of 2.93 (95% CI, 1.24–6.91) (Fig. 2A). Causes of death were available in 9 (48%) deaths. Among cases, causes were: arthropathy (or related disorder), thyroid cancer, prostate cancer, and pneumonia. Amongst controls, the causes were external/unspecified event (x2), circulatory system disorder, COPD, lung cancer, aortic valve disorder, and senility. Under the analyses using the most conservative definition of likely or highly likely grading only, 4 (6.5%) cases died during a median follow-up of 7.3 (IQR, 3.0–12.4]) years, at a median age of 61 [IQR, 56–66]. Three (1.2%) controls died during a median follow-up of 6.0 (IQR, 2.6–11.0) years, at a median age of 68 [IQR, 29–71], yielding a hazard ratio of 6.65 (95% CI, 1.44–30.72) (Fig. 2B).

For the sensitivity analysis in which follow-up was extended until end of study period (irrespective of transfers out of practice and last collection date) for the sample of 535 patients in England and Wales, a total of 23 deaths occurred (8 cases and 15 controls), yielding a hazard ratio of 2.88 (95% CI, 1.18–7.00); *P* = 0.020.

## Discussion

This nationally representative study provides for the first time an estimated prevalence of XLH in both childhood and adulthood, with 2016 estimates for these being 15.1 (95% CI, 11.3–20.1) per million and 15.7 (95% CI, 11.8–20.9) per million, respectively. We observed an unexpected reduction in survival among XLH cases relative to controls (with average age at death being approximately 8 years younger in cases relative to controls), which was evident when using either the more or less conservative case definition.

Three studies have previously estimated the prevalence of XLH, although these should be cautiously compared with the present investigation due to differences in study populations and methods used. Most recently, 21 children were identified with hereditary rickets and confirmed genetically from pediatric hospitals in Norway in 2009 (12), giving a prevalence of 1 in 60 000 (16.6 per million) among individuals from birth to18 years of age. These children were also identified using the ICD 10 code E83.3 for disorders of phosphorus metabolism and phosphatases in the Norwegian Patient Register, a nationwide health administrative registry after discounting hypophosphatasia and other causes such as hyperparathyroidism, renal tubular disorders such as Fanconi, and vitamin D deficiency. A Danish study used hospital medical records and ICD8/10 codes for rickets to identify patients referred or discharged over a 20-year period in Southern Denmark (10), and reported a prevalence of 4.8 per 100 000 (48 per million) among those from birth to 14.9 years of age. This estimate was based on 15 cases with XLH, but excluded patients aged 15 years or older and so did not estimate adult prevalence or outcomes. The medical records were reviewed to exclude rickets from renal insufficiency, renal tubular acidosis, liver/biliary disease, malabsorption, related syndromes, or iatrogenic causes. If cases with suspected nutritional rickets had a serum 25-hydroxy vitamin D of more than 50 nmol/L they were also excluded. Biochemical criteria included: raised serum ALP, low serum phosphate, and a normal serum calcium. In Japan, a survey was used to ascertain the numbers of patients with FGF23-related disorders from 2005 to 2009 (11). Forty-one patients with genetic hypophosphatemic diseases (63% female) were identified and the incidence of XLH was estimated to be 1 per 20 000 live births (50 per million). Inclusion and exclusion criteria for cases were not described nor was the potential bias from a nonrandom response rate considered. There remains inconsistency in the methods for case ascertainment across studies and further collaborative work is needed to standardize procedures for case validation and selection of controls.

Prevalence estimates in the current analysis generally increased over the study period (1995–2016), although this was more marked among adults and in all possible cases vs the more conservative definition (likely and highly likely). The secular trend is most likely driven by changes in clinical practice for laboratory testing, documentation and quality of coding during this time. For instance, among all (522) potential XLH cases, the proportion with no serum phosphate testing declined during the study period from 54% (1995–1999) to 42% (2012–2016) (results not shown), while for serum alkaline phosphatase this decline was from 34% to 27% for the same years.

The mechanism for the observed increase in mortality in adults with XLH is not known. It could be that the reduced survival is indirect, driven by an imbalance in comorbidities and other associated characteristics of XLH patients relative to controls. Furthermore, a difference in the management of comorbidities between cases and controls may also exist. A direct FGF23 pathway is also possible. It has previously been shown that neutralizing circulating FGF23 reverses several key biochemical and musculoskeletal features of XLH in both animals (18) and humans (8, 9), suggesting a key role of FGF23 in XLH. Since its discovery (19), various data have been submitted to support an association between elevated FGF23 and a number of common diseases (20–22) and mortality (23), although further work is needed to confirm and/or further elucidate these findings. For instance, changes in cardiac size have not been seen in *hyp* mice up to the age of 30 weeks (24), and neutralizing FGF23 a) did not prevent cardiac hypertrophy in rats that underwent subtotal nephrectomy, and b) increased mortality, this may reflect different effects of FGF23 blockade based on the underlying mechanisms of excess (25). The direct vs indirect role of FGF23 in vascular calcification remains controversial (26, 27). Untangling a potential direct pathogenic role for FGF23 is relevant for patients with XLH, as the current therapies for this rare disease include phosphate and activated vitamin D supplementation, which are associated with increases in FGF23 in both adults and children (28) and worsening enthesopathy in mice (29). The consequences of FGF23 on survival in other rare bone diseases that involve FGF23, such as fibrous dysplasia (30) and cutaneous skeletal hypophosphatemia syndrome (31), are also unknown.

In XLH, it is likely that the FGF23 pathway may not be the only molecular mechanism perturbed. In the absence of an XLH-specific scale, little correlation has been found between broad measures of phenotype severity, such as presence of bowing and dental abscesses, with the type or location of genetic mutation between individuals or even within families (32). These findings suggest a possible involvement of other genetic and environmental factors in determining the clinical phenotype of individuals with XLH; although whether these are also implicated in the observed reduction in survival is unknown. Future work will seek to further elucidate the findings from our study, including analyses to better ascertain the natural history of XLH in terms of the burden of comorbidity, therapies and associated medical procedures using secondary care data, which will also likely offer insight into the elevated mortality rate here observed.

### Strengths and limitations

This is the first study to examine the prevalence and prognosis of XLH in adulthood. A major strength is the generalizability of the findings which arise from a population-based, real-world dataset having linkage to gold-standard death registry data and by taking a conservative approach to case identification with 2 methods for case ascertainment. The specificity that these cases represent patients with XLH is supported by the observed excess of females expected from an X-linked disorder, even though graders were blinded to sex status of potential cases. Another strength is the ability to identify relevant controls that allowed the determination of outcomes attributable to XLH.

A major limitation is that no validated algorithm exists for confirming the diagnosis of XLH in the primary case setting. The potential for miscoding rare disease is also likely higher in the primary care setting where coding is completed by a clinician, nurse, or administrative staff with little or no training in rare diseases; this was highlighted by the responses from the GP verification questionnaires, where cases were found to be discordant to expert clinical grading (results in Supplementary Item 7) (16). Given the lack of genetic and radiological data in the primary and secondary care datasets and restrictions to directly contact patients, we were unable to genotype identified cases for the *PHEX* mutation or check their radiological findings (the gold standards for case validation). Furthermore, we omitted diagnosis of Dent’s disease as a potential mimicking disease in the initial extraction, which meant 11 patients subsequently identified in CPRD GOLD using the Read code for Dent’s disease were not considered. Read codes for hypercalciuria and renal stones identified only 4 of 122 (3.3%) adjudicated cases (compared to 17/400 [4.3%] in matched controls), of whom only 2 (1.6%) (vs 16/400 [4.0%] of controls) had such a diagnosis preceding the date of potential XLH diagnosis. Future algorithms should consider Dent’s disease in the differential diagnosis of XLH.

In addition, the Read coding system does not include coding for family history of XLH that would further support the diagnoses. Due to information governance, analysis of free text in the clinical or radiological reports was not possible, which may have helped by describing known radiological features of XLH such as mineralizing enthesopathy. Another limitation is that by taking a very conservative approach to case identification, we have likely underestimated the prevalence in exchange for specificity that was required for the matched analysis for prognosis. Even after calculation of missing laboratory tests, a number of potential participants were still unable to be graded because of missing laboratory values (Supplementary Item 6) (16). As laboratory tests performed in the secondary care setting are not included in CPRD GOLD, the prevalence of young children, who are unlikely to have blood tests in primary care would have been underestimated. Similarly, given there is no agreed guideline for the routine care of adults with XLH, incorporating frequency of laboratory testing and treatment with activated vitamin D and phosphate supplements into our case adjudication process may have missed patients with XLH, leading to a conservative prevalence of XLH. Were this to be the case, it may have led to relatively severe cases being favored for inclusion, which may have subsequently impacted the generalizability of mortality estimates. However, it is worth noting that main findings remained unchanged in a post hoc sensitivity analysis comparing all 522 potential XLH cases with their controls (mortality hazard ratio of 2.87 [95% CI, 2.08–3.95]; *P* < 0.001). Linked ONS data were only available for England and Wales and the linkage also depended on local practice agreements being in place, so only 60% of practices in total were linked, although CPRD mortality data have previously been shown to be reliable (irrespective of ONS linkage) (14). 

## Conclusion

We describe conservative estimates of XLH prevalence in both childhood and adulthood. We find a concerning reduction in survival in adults that has not been previously reported and which may have implications for other FGF23-related disorders.

## Abbreviations

ALP: alkaline phosphatase
CPRD: UK Clinical Practice Research Datalink
FGF23: fibroblast growth factor 23
GP: general practitioner
IQR: interquartile range
ONS: Office for National Statistics
XLH: X-linked hypophosphatemia

## Additional Infromation

***Disclosure Summary:*** Kyowa Kirin International provided funding for this project to the University of Oxford. Outside the submitted work, SH, NS, and AD report no conflicts of interest. DPA reports grants from AMGEN, UCB Biopharma, and Les Laboratoires Servier. CC reports personal fees from Alliance for Better Bone Health, Amgen, Eli Lilly, GSK, Medtronic, Merck, Novartis, Pfizer, Roche, Servier, Takeda, and UCB. RPV has received research grants from Kiowa Kirin, and consultancy fees outside the scope of this study. MKJ reports grants from AMGEN, Kyowa Kiran Hakin, and consultancies from AMGEN, Internis, consilient Health, Mereo Biopharma, Kyowa Kirin Hakin and UK

***Data Availability:*** Restrictions apply to the availability of data generated or analyzed during this study to preserve patient confidentiality or because they were used under license. The corresponding author will on request detail the restrictions and any conditions under which access to some data may be provided.
